# Effect of Dietary Sodium and Potassium Intake on the Mobilization of Bone Lead among Middle-Aged and Older Men: The Veterans Affairs Normative Aging Study

**DOI:** 10.3390/nu11112750

**Published:** 2019-11-13

**Authors:** Xin Wang, Douglas Kim, Katherine L. Tucker, Marc G. Weisskopf, David Sparrow, Howard Hu, Sung Kyun Park

**Affiliations:** 1 Department of Epidemiology, School of Public Health, University of Michigan, Ann Arbor, MI 48109, USA; xwangsph@umich.edu (X.W.); doug92kim@gmail.com (D.K.); 2 Department of Clinical Laboratory and Nutritional Sciences, University of Massachusetts at Lowell, Lowell, MA 01854, USA; katherine_tucker@uml.edu; 3 Department of Environmental Health, Harvard T.H. Chan School of Public Health, Boston, MA 02115, USA; mweissko@hsph.harvard.edu; 4 Normative Aging Study, Veterans Affairs Boston Healthcare System, and Department of Medicine, Boston University School of Medicine, Boston, MA 02118, USA; David.Sparrow@va.gov; 5 School of Public Health, University of Washington, Seattle, WA 98195, USA; hhu5@uw.edu; 6 Department of Environmental Health Sciences, School of Public Health, University of Michigan, Ann Arbor, MI 48109, USA

**Keywords:** sodium, potassium, patella lead, tibia lead, urinary lead, bone lead resorption, middle-aged and older men

## Abstract

Bone is a major storage site as well as an endogenous source of lead in the human body. Dietary sodium and potassium intake may play a role in the mobilization of lead from bone to the circulation. We examined whether association between bone lead and urinary lead, a marker of mobilized lead in plasma, was modified by dietary intake of sodium and potassium among 318 men, aged 48–93 years, in the Veterans Affairs (VA) Normative Aging Study. Dietary sodium and potassium were assessed by flame photometry using 24-h urine samples, and a sodium-to-potassium ratio was calculated from the resulting measures. Patella and tibia bone lead concentrations were measured by K-shell-x-ray fluorescence. Urinary lead was measured by inductively coupled plasma mass spectroscopy in 24-h urine samples. Linear regression models were used to regress creatinine clearance-corrected urinary lead on bone lead, testing multiplicative interactions with tertiles of sodium, potassium, and sodium-to-potassium ratio, separately. After adjustment for age, body mass index, smoking, vitamin C intake, calcium, and total energy intake, participants in the highest tertile of sodium-to-potassium ratio showed 28.1% (95% CI: 12.5%, 45.9%) greater urinary lead per doubling increase in patella lead, whereas those in the second and lowest tertiles had 13.8% (95% CI: −1.7%, 31.7%) and 5.5% (95% CI: −8.0%, 21.0%) greater urinary lead, respectively (*p*-for-interaction = 0.04). No statistically significant effect modification by either sodium or potassium intake alone was observed. These findings suggest that relatively high intake of sodium relative to potassium may play an important role in the mobilization of lead from bone into the circulation.

## 1. Introduction

Lead toxicity is a prevalent and persistent public health problem. People have been commonly exposed to lead through various routes including air, dust, paint, water, and food [1]. In recent years, the public health importance of lead toxicity has attracted intense interest, due to large-scale lead exposure events such as in Flint, Michigan, with poisoning due to lead in the water. This has taken place in more than 3000 U.S. neighborhoods, with lead poisoning rates in some even greater than those seen in Flint [2]. For the other exposure routes, primary prevention strategies such as removal of lead from gasoline and elimination of lead solder from food and beverage cans have reduced environmental sources of lead remarkably [3]. However, lead stemming from such environmental exposures remain in the body, specifically within bones, for decades, leading to adverse health impacts in aging populations [4]. Low-to-moderate chronic exposure to lead has been associated with age related conditions such as cognitive decline, hearing loss, kidney failure, and cardiovascular disease [5,6,7,8]. Recently, low-level environmental lead exposure has been further recognized as a largely overlooked risk factor for mortality, especially from cardiovascular disease, in the United States [9]. It has been estimated that of the 2.3 million deaths every year in the U.S., about 412,000 can be attributable to lead exposure [9].

Once lead has entered the body from external environmental exposure, there are several possible fates and pathways. Initially, the blood serves as the receptacle of absorbed lead and more than 99% of lead is bound to erythrocytes [10]. However, less than 2% of blood lead is free in plasma, and is the most biologically active fraction of lead in circulation, which can enter peripheral target tissues (e.g., brain, kidney, skeleton) causing organ toxicity [11,12,13,14]. The circulatory lead can also be deposited into various bone sites. In adults, more than 95% of the total lead body burden is found in the skeleton [4]. Lead enters bone at the time of mineral deposition with the half-life in the order of years to decades, and will then be mobilized from the bone at the time of bone resorption [15,16]. Bone lead is, therefore, considered as an ideal biomarker for characterizing cumulative lead exposure. It is also an important source of endogenous lead exposure in individuals with a history of high-level occupational or environmental lead exposure, even after the environmental exposure has been reduced. Previous studies have shown that bone lead independently contributes to plasma lead, and this can be modified by the bone resorption rate [15,17]. Given that an increase in bone resorption is a characteristic of aging, release of bone lead into the plasma is considered as an important source of lead toxicity in older adults [18]. This is supported by the superiority of bone lead over whole blood lead in predicting aging-related chronic health outcomes such as hypertension, coronary heart disease, and mortality [8,19,20].

The role of dietary micronutrients in lead metabolism has specifically been of interest as they pose a logistically simple intervention to prevent lead toxicity due to bone lead resorption. Dietary factors affecting bone turnover such as sodium and potassium may be associated with the mobilization of lead from bone into the circulation. Low potassium and high sodium dietary intake have each been reported to be associated with accelerated bone resorption in both animal and population-based studies [21,22,23]. A recent cross-sectional study reported that urinary sodium-to-potassium (Na/K), but not individual values of sodium or potassium, were inversely related to bone mineral density (BMD) [24]. To our knowledge, however, there is little information currently available regarding the role of sodium and potassium on lead metabolism, specifically bone lead resorption, in older populations.

**Figure 1:**
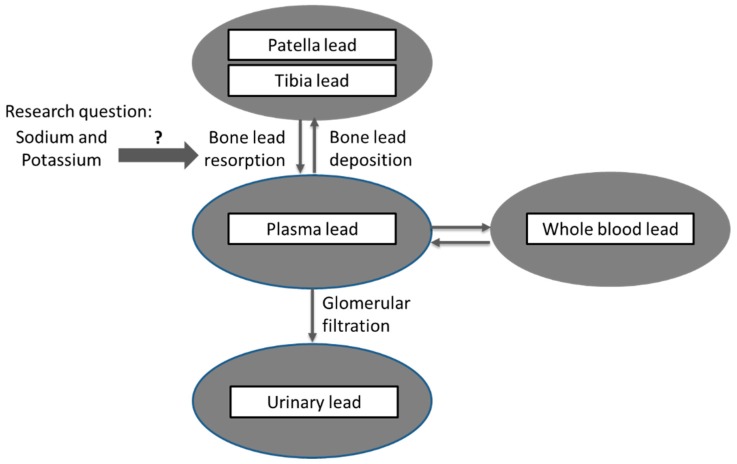
Conceptual model of the current study.

To address this issue, we examined associations between markers of dietary sodium and potassium intakes, measured from 24-h urine collection, which is considered more accurate measures of dietary intake [25], and bone lead resorption in the VA Normative Aging Study (NAS), a prospective cohort of community-dwelling middle-aged to elderly men. Although plasma lead provides a toxicologically available fraction of circulatory lead, it is difficult to measure accurately due to its low concentration and possible contamination from laboratory handling [15,17]. Urinary lead originates from plasma lead that has been filtered at the glomerular level. Thus, 24-h urinary lead adjusting for glomerular filtration rate has been an alternative to measuring plasma lead [15,17]. In this study, we aimed to assess the effect modification by dietary intake of sodium and potassium as well as Na/K ratio, on the association between bone lead concentration and 24-h urinary lead excretion (Figure 1). We hypothesized that higher sodium intake and/or lower potassium intake would increase the release of lead from bone.

## 2. Materials and Methods

### 2.1. Ethics

All participants provided written informed consent. This study was reviewed and approved by the Institutional Review Boards of each participating institute: the University of Michigan School of Public Health, Harvard School of Public Health, and the Department of Veterans Affairs Boston Healthcare System. Ethical approval number is HUM00026596. 

### 2.2. Study Population

Participants in the current analysis were from the Normative Aging Study, a prospective cohort of community-dwelling middle-aged to elderly men with no known occupational lead exposure over time. Between 1961 and 1962, a total of 2280 men from Boston, Massachusetts participated. They were predominantly non-Hispanic white and ranged in age from 21 to 80 years upon enrollment. All participants were free of past or present chronic medical conditions including heart disease, cancer, diabetes, peptic ulcer, gout, bronchitis, sinusitis, recurrent asthma, or hypertension at the time of enrollment. They returned for an examination every three to five years for a detailed core examination including the collection of medical history, routine physical examination, laboratory tests, dietary intake, and other factors that might influence health.

**Figure 2:**
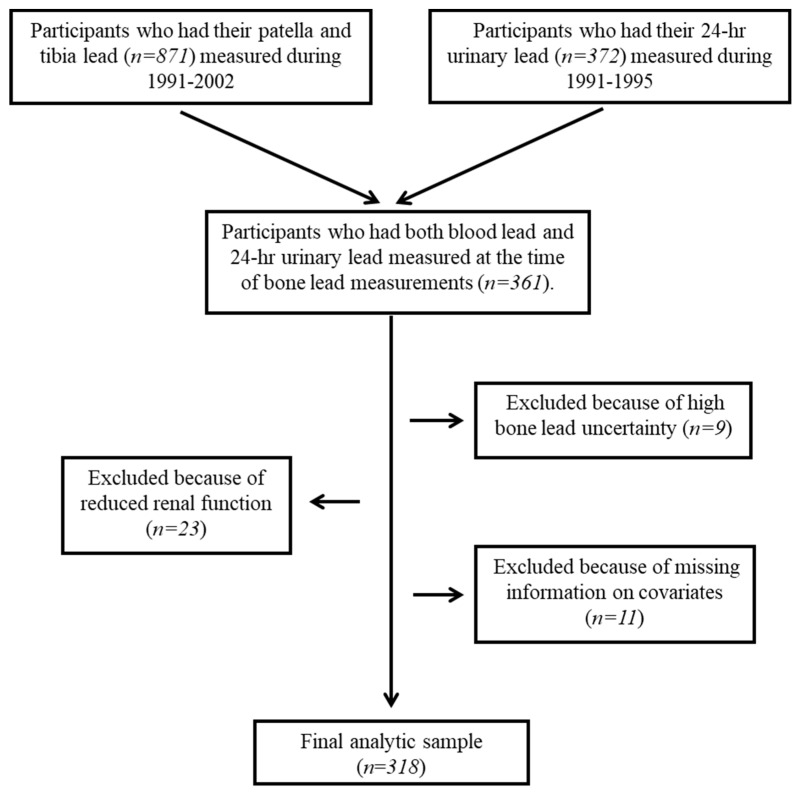
Schematic diagram of the study methodology of the analytic sample in the Veterans Affairs Normative Aging Study.

Between August 1991 and August 2002, 871 consecutive NAS participants underwent patella and tibia bone lead measurements using K-shell-x-ray fluorescence (KXRF). Between June 1991 and April 1995, 372 participants had their 24-h urine samples collected for urinary lead, sodium, and potassium concentration determinations. In addition, a blood sample for blood lead analysis was collected at each visit since 1988. For the present study, we identified 361 NAS participants who participated in the KXRF bone lead sub-study and who had 24-h urinary lead, sodium, potassium, and blood lead concentrations measured at the same visit as the bone lead measurements. We excluded nine participants with high bone lead concentration uncertainties, 23 participants with reduced renal function (serum creatinine concentration ≥ 1.5 md/dL), and 11 participants with missing information on key covariates (body mass index, smoking, dietary calcium and vitamin C intake, and total energy intake), leaving a final sample of 318 men for the analyses (Figure 2).

### 2.3. Blood Lead and Bone Lead Measurements

Whole blood samples were collected in trace metal-free tubes containing ethylenediaminetetraacetic acid and analyzed to obtain blood lead concentration by graphite furnace atomic absorption spectroscopy (ESA Laboratories, Chelmsford, MA, USA). Detailed measurement procedures are described elsewhere [26]. The limit of detection (LOD) of blood lead in NAS was 1 μg/dL. For the five participants in our study sample with a blood lead concentration below the LOD (detection rate = 98.4%), a value equal to 1/√2 μg/dL was imputed.

NAS participants had bone lead concentrations measured for 30 min each at the patella and mid-tibial shaft, using the ABIOMED KXRF instrument, as described in detail previously [27]. Analysis of lead in both bone sites provides an index of cumulative lead exposure in humans. The patella is primarily made up of trabecular bone while the tibia contains more cortical bone. Lead in trabecular bone is more prone to bone turnover, with a half-life of a few years, compared to lead retention in cortical bone, which has a half-life of decades. Therefore, in general, tibia bone lead has been viewed as an indicator of lifetime cumulative exposure, while patella bone lead has been identified as an endogenous lead reserve, more closely related to lead in circulation [17,28]. The KXRF instrument provides an unbiased estimate of bone lead concentration in μg/g bone mineral, with an accompanying uncertainty estimate related to background noise in the signal extraction procedure. Participants with uncertainty estimates beyond the typical range (>10 and >15 μg/g for tibia and patella, respectively) were further excluded, as these estimates usually reflect excessive movement during the measurement.

### 2.4. Urinary Lead, Sodium, and Potassium Measurements

Detailed measurement procedures for urinary lead, sodium, and potassium were described elsewhere [17,29]. In brief, a week before the scheduled NAS regular medical examination, the participant received a 4-L container by mail for 24-h urine collection. Each container came with instructions for use and a questionnaire about the collection process including time of collection, medicine use, spillage, and missed collections. Twenty-four hour urine collection began after the first void of the morning and continued through the first void of the subsequent morning. Urine samples were collected in trace metal-free tubes containing sodium metabisulfite and 6 M hydrochloric acid, with a pH between 2 and 3. Aliquots of the urine samples were frozen at −20 °C; and thawed and digested with nitric acid at room temperature at the time of assay. Urinary lead concentration was analyzed with high-resolution inductively coupled plasma-mass spectrometry (ICP-MS) (Sciex Elan 5000; Perkin Elmer, Norwalk, CT, USA). Urinary sodium and potassium concentrations were determined by emission flame photometry. Twenty-four hour urinary excretions of lead, sodium and potassium were calculated as analyte concentration × urinary volume, corrected to 24 h.

### 2.5. Covariates

Covariates were chosen a priori and included age, smoking status, body mass index (BMI), and dietary vitamin C and calcium intake based on previous studies of factors predicting lead concentrations [30,31]. Age and smoking status were obtained via self-administered questionnaires at each NAS regular visit. Cigarette smoking was categorized as current vs. former vs. never smoking. BMI was calculated as weight (kg)/height (m^2^) obtained during physical examination. To assess the dietary intake, we used a validated, semi-quantitative food frequency questionnaire adapted from the one used in the Nurses’ Health Study that inquired about the average consumption of 135 food items during the past year [32,33,34]. Dietary calcium, vitamin C, and total energy intake were computed from the reported frequency of consumption of each specified unit of food and from published data on the nutrient content of the specified portions [34]. Creatinine in serum and 24-h urine samples were determined by a Jaffe rate reaction with a Beckman Creatinine Analyzer 2 (Beckman, Brea, CA, USA). Urinary N-telopeptide (NTx) was analyzed with a commercially available competitive-inhibition enzyme-linked immunosorbent assay (Osteomark; Ostex International, Seattle, WA, USA) and was expressed as creatinine corrected bone collagen equivalents (nM BCE/mM creatinine).

### 2.6. Statistics

Univariate statistics were calculated and examined for each tertile of the Na/K ratio (low: <2.2; medium: 2.2–3.0; high: >3.0). Twenty-four hour urinary excretion of lead was adjusted for creatinine clearance rate (CCr), an estimate of glomerular flow rate calculated from the clearance of endogenously produced creatinine, to account for the variability of glomerular function between participants. CCr (mL/min) was calculated as the total amount of urine creatinine over 24 h (mg)/[serum creatinine concentration (mg/mL) × collection time (min)], which represents the volume of blood plasma that is cleared of creatinine per unit time. Creatinine clearance-corrected urinary lead excretion was calculated using the residual method, where residuals from a linear regression model with log-transformed urinary lead as the dependent variable and CCr as the independent variable were added to a constant, which was equal to the value for predicted urinary lead when CCr was at the mean value of the study population. To examine the influence of dietary sodium and potassium on the mobilization of lead into the circulation, we regressed creatinine clearance-corrected urinary lead on bone/blood lead as well as with interaction terms indicating tertiles of sodium, potassium, and Na/K ratio, separately, in linear regression models. Age, BMI, smoking status, vitamin C intake, calcium intake, and total energy intake were adjusted in all models. Given the highly skewed distributions of urinary, blood, and bone lead concentrations, logarithmic transformations with base 2 were applied to all lead measures; dose-response relationships were closer to linear after log-transformation. We chose base 2 rather than natural log-transformation for easier interpretation of the regression coefficients. Effect estimates and 95% CIs were expressed as percent change in 24-h urinary lead excretion per 2-fold increase in patella, tibia, and blood lead concentrations.

In light of previous evidence from the NAS cohort suggesting that bone resorption significantly modifies release of lead from bone [15], we further examined whether the influence of dietary sodium and potassium intake on bone lead resorption could be through its impact on the bone resorption rate by additional adjustment for multiplicative interaction terms between urinary NTx concentration and bone lead in all regression models. All analyses were conducted with SAS, version 9.4 (SAS Institute Inc., Cary, NC, USA).

## 3. Results

Characteristics of the study population by tertiles of Na/K ratio are listed in Table 1. Participants had a mean age of 66.6 years (SD = 7.0 years), ranging from 48 to 93 years. The mean 24-h urinary excretions of sodium and potassium were 133 mmol/day (SD = 61.2 mmol/day) and 53.0 mmol/day (SD = 23.1 mmol/day), respectively, with the mean Na/K ratio equal to 2.7 (SD = 1.1). Men with higher Na/K ratio were more likely to have higher BMI and lower dietary calcium and vitamin C intake. Smoking status differed significantly across the Na/K ratio categories (*p* < 0.0001). Geometric mean concentration of patella lead was lowest in men in the first tertile of the Na/K ratio (*p* = 0.04). No significant differences in urinary, tibia, or blood lead concentrations across the Na/K tertiles were observed.

**Figure 3:**
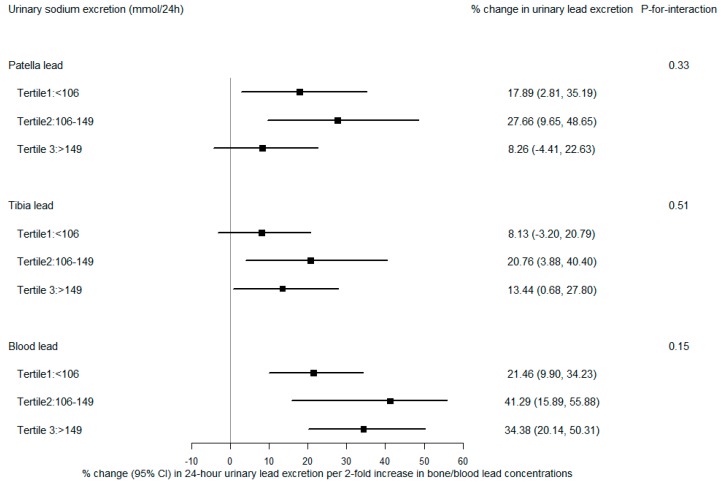
Percent change in 24-h urinary lead excretion (µg/24 h) per 2-fold increase in patella, tibia, and blood lead concentrations, stratified by tertiles of 24-h sodium excretion. All models were adjusted for age, body mass index, smoking, 24-h potassium excretion, vitamin C intake, calcium intake, and total energy intake.

**Figure 4:**
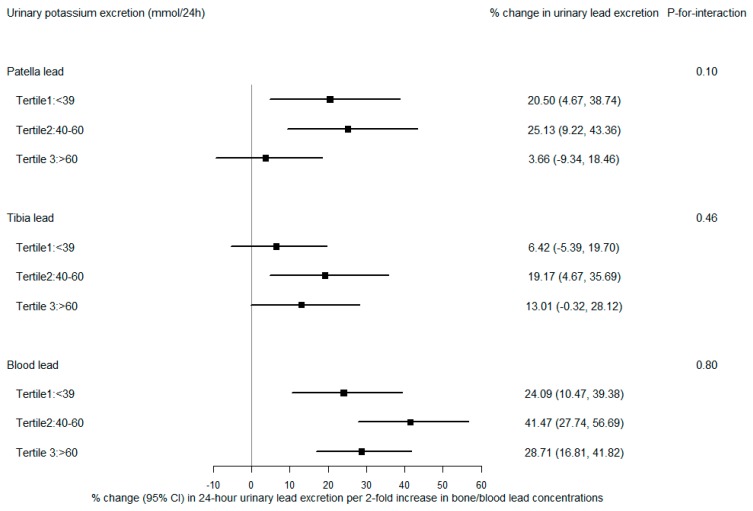
Percent change in 24-h urinary lead excretion (µg/24 h) per 2-fold increase in patella, tibia, and blood lead concentrations, stratified by tertiles of 24-h potassium excretion. All models were adjusted for age, body mass index, smoking, 24-h sodium excretion, vitamin C intake, calcium intake, and total energy intake.

Figure 3 and Figure 4 show associations of urinary lead with patella lead, tibia lead, and blood lead stratified by tertiles of sodium and potassium excretion, respectively. Patella lead was more strongly associated with urinary lead in the lower two tertiles of potassium excretion than in the highest tertile, where a borderline significant effect modification (*p*-for-interaction = 0.10) was observed (Figure 4). After adjusting for age, BMI, smoking, sodium excretion, vitamin C intake, calcium intake, and total energy intake, participants with low and medium potassium excretion had mean 20.5% (95% CI: 4.7%, 38.7%) and 25.1% (95% CI: 9.2%, 43.4%) increases in 24-h urinary lead excretion (µg/24 h) per doubling increase in patella lead concentration, respectively, while those with high potassium excretion had only a 3.7% (95% CI: −9.3%, 18.5%) increase in urinary lead excretion. There was no statistically significant influence of sodium or potassium intake on the associations of urinary lead with tibia lead and blood lead.

**Figure 5:**
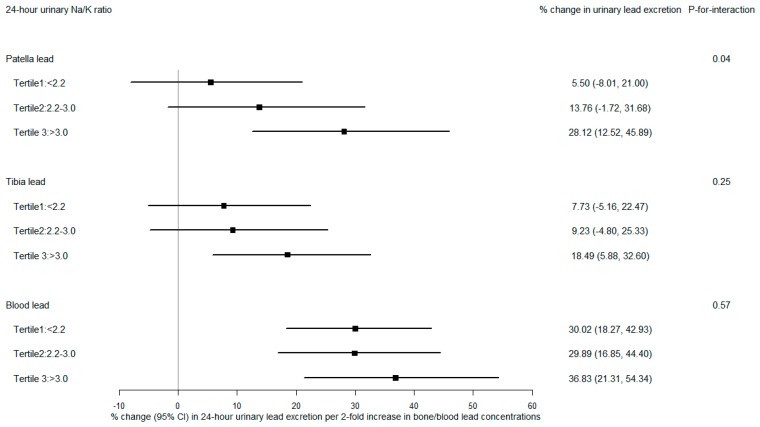
Percent change in 24-h urinary lead excretion (µg/24 h) per 2-fold increase in patella, tibia, and blood lead concentrations, stratified by tertiles of 24-h sodium-to-potassium (Na/K) ratio. All models were adjusted for age, body mass index, smoking, vitamin C intake, calcium intake, and total energy intake.

A significant modifying effect of Na/K ratio on the relationship between urinary lead and patella lead was observed (Figure 5). The relation of patella lead to urinary lead was greater in participants with higher Na/K ratio; those in the highest tertile showed a 28.1% (95% CI: 12.5%, 45.9%) increase in urinary lead per doubling increase in patella lead, whereas those in the second and lowest tertiles had a 13.8% (95% CI: −1.7%, 31.7%) and 5.5% (95% CI: −8.0%, 21.0%) increase in urinary lead, respectively (*p*-for-interaction = 0.04). Similarly, stronger associations between urinary lead and tibia lead were observed in higher Na/K ratio tertiles. However, the interaction between tibia lead and Na/K ratio was less pronounced and not statistically significant (*p*-for-interaction = 0.25). Again, we found no statistically significant effect modification by Na/K ratio on the relationship of blood lead to urinary lead.

The effect modification by Na/K ratio on the association between urinary lead and bone lead, after further incorporation of blood lead as a covariate in the regression models, is shown in Supplementary Figure S1. As expected, the magnitude of the relationship of bone lead to urinary lead decreased, but the pattern of the associations across Na/K ratio tertiles remained unchanged. For example, those in the highest Na/K ratio tertile showed a mean 14.3% (95% CI: 1.2%, 29.0%) increase in urinary lead per doubling increase in patella lead, whereas those in the medium and low tertiles had weaker associations (*p*-for-interaction = 0.04).

In models with further adjustment for NTx and its cross-product term with patella/tibia lead, attenuated modifying effects of Na/K ratio on the relationships of both patella lead (*p*-for-interaction = 0.08 in Supplementary Figure S2 vs. *p*-for-interaction = 0.04 in Figure 5) and tibia lead (*p*-for-interaction = 0.32 in Supplementary Figure S2 vs. *p*-for-interaction = 0.25 in Figure 5) to urinary lead were observed.

## 4. Discussion

Bone is the major storage site as well as endogenous source of lead in the human body [4,15]. The release of bone lead into the circulation is an important source of soft-tissue lead exposure and toxicity in older populations [5,6,7,8,18]. Dietary strategies provide the possibility of effective and affordable ways to deal with lasting adverse health effects related to bone lead resorption [32]. To the best of our knowledge, this is the first study to evaluate the influence of dietary sodium and potassium intake on the release of lead from bone. Bone lead was found to be positively associated with urinary lead after adjustment for CCr. Moreover, we observed a stronger relation of patella lead to urinary lead among the participants with both higher sodium intake and lower potassium intake (higher Na/K ratio). The observed effect modification remained significant after adjusting for age, BMI, smoking, vitamin C intake, calcium intake, and total energy intake. These findings suggest that a relatively high intake of sodium relative to potassium may play an important role in the mobilization of lead from bone into the circulation in aging populations. However, our findings need to be interpreted with a caution given the limited power.

Few studies have been conducted to examine the associations between dietary micronutrient intakes and lead biomarkers directly. One cross-sectional study of pregnant women in South Korea revealed that maternal sodium intake was positively associated with blood lead concentration during the pregnancy [35]. This finding raised the concern that dietary sodium intake might play a role in the release of lead from bone stores during pregnancy, a period of increased bone resorption, through its adverse effects on bone metabolism. However, neither bone lead nor urinary lead was examined in this Korean study [35]. In our study, we did not find a significant effect modification by sodium intake or potassium intake alone on the association between urinary lead and bone lead. Instead a significant increase in the relation of patella lead to urinary lead was detected in participants with a high intake of sodium relative to potassium. This suggests that the ratio of sodium and potassium, rather than either sodium or potassium individually, may be a more important determinant of bone lead resorption in populations with accelerated bone resorption.

Previous studies in the NAS cohort have demonstrated that bone resorption rate significantly influenced the release of bone lead stores into circulation [15,17]. In the current study, additional adjustment of NTx and its cross-product term with patella/tibia lead also attenuated the modifying effects of Na/K ratio on the relation of bone lead to urinary lead, suggesting that a high intake of sodium relative to potassium may affect bone lead resorption, partly through its impact on general bone resorption. Epidemiologic studies have shown that higher urinary sodium was associated with higher calcium excretion and bone resorption markers, lower BMD, and greater risk of osteoporosis [22,36,37,38]. In animal models, rats with higher levels of supplemented sodium showed reduced bone calcium content, accelerated bone loss due to elevated bone resorption, increased urinary calcium and hydroxyproline excretion as well as increased levels of serum parathyroid hormone and urinary cyclic adenosine monophosphate [39,40,41,42,43,44]. On the other hand, a high potassium diet has been reported to be favorable for the maintenance of bone mass, especially in middle-aged to older people [23,45,46,47]. Both human and in vivo evidence suggests that maintenance of a mild metabolic alkalosis, with the anion-independent effect on calcium excretion and bone metabolism, might be a potential mechanisms underlying the beneficial effects of a higher potassium intake on bone including improved calcium and phosphorus balance, improved BMD, reduced bone resorption, and increased bone formation [48,49,50,51]. In addition to low potassium intake or sodium excess, a disproportionate intake of sodium compared to potassium has previously been noted to adversely affect bone metabolism. In a randomized trial of 60 postmenopausal women who had adapted to a low-salt diet (87 mmol/day sodium) for three weeks, those assigned to a 4-week high-salt (225 mmol/day sodium) diet plus potassium citrate (90 mmol/day) showed significantly lower NTx and urinary calcium excretion when compared to participants assigned to high-salt diet plus placebo [52]. More recently, a community-based cross-sectional study of 3265 middle-aged to older men and women found that the Na/K ratio, but neither sodium or potassium alone, was inversely associated with the BMD of the whole body and the majority of hip sites [24]. These findings could be explained, in part, by the interactions between sodium and potassium in the maintenance of calcium homeostasis in the kidney. Urinary sodium and calcium share a common transport mechanism in the proximal tubule and loop of Henle of the kidney [53]. A high dietary sodium intake increases urine calcium excretion and influences calcium homeostasis as a result of decreased calcium reabsorption by stimulating reabsorption of filtered sodium by the sodium-hydrogen exchanger 3 (NHE3) [24,54]. A high potassium intake, on the other hand, decreases sodium reabsorption in the proximal tubule and in the loop of Henle by suppressing NHE3 through the rennin-angiotensin system, which in turn, promotes calcium retention even in the setting of high sodium intake [23,49,55,56,57]. Additionally, potassium has also been found to reduce the extracellular volume expansion that occurs with increased sodium intake, which may be partly responsible for the decreased calcium excretion observed in people with a high salt diet [52].

We did not detect a statistically significant modifying effect of Na/K ratio on the relationship of tibia lead to urinary lead. A possible explanation for the discrepancy in the results between patella and tibia lead is the distinct bone composition. The patella is composed mostly of trabecular bone, which has a higher resorption rate; in contrast, the tibia constitutes more cortical bone with a slow rate of bone turnover and a longer half-life [58]. Trabecular bone typically has more active metabolism than cortical bone [59]. Hence, lead in trabecular bone is more available for mobilization than that in cortical bone. This has been supported by stronger associations of trabecular bone lead with circulating lead in previous studies [12,15,17,60]. In this way, it is expected that the resorption of patella lead may be more influenced by lifestyle factors, especially dietary factors, than tibia lead [32].

Our findings suggest dietary modifications targeted at decreasing bone lead resorption may further translate to reductions in the risk of chronic diseases related to lead exposure from endogenous sources, particularly in aging populations. Bone lead concentrations have been strongly associated with incidence of hypertension and coronary heart disease in the NAS cohort [8,19]. Low-level lead exposure has also been identified as an important risk factor for cardiovascular disease mortality in the U.S. in one recent prospective study using the data from the National Health and Nutrition Examination Survey III (1988–1994), in which the lead measured in blood is more likely to be a reflection mostly of lead mobilized from bone, rather than on-going environmental exposure [9]. Feasibility and effectiveness of efforts to reduce the lead resorption in the prevention of lead-related diseases should be addressed in more research in the future. Our finding may also suggest that lead exposure should be considered in the epidemiologic studies focusing on health outcomes associated with an excess of sodium and/or a deficit of potassium, for example, hypertension [61], in which the release of lead from bone may play an important role. Important strengths of this study include the utilization of 24-h urinary concentrations of sodium and potassium, instead of the more commonly used measures derived from food frequency surveys, allowing for a more accurate assessment of dietary sodium and potassium intakes in participants [25]. Additionally, bone lead concentration was measured. Unlike blood lead concentration, which reflects only recent exposure to lead, bone lead is an endogenous reservoir of body lead and captures long-term lead exposure during a person’s lifetime [4,15]. Previous studies have demonstrated that blood lead not only inadequately represents lead accumulated in bone, but also inadequately represents bioavailable lead in plasma [15,17]. Furthermore, bone lead has been found to be an independent and more closely related contributor to plasma lead [15,17].

Nevertheless, our study has several limitations. First, the cross-sectional nature of our study sample precludes the ability to determine chronicity among different lead measurements and dietary factors. Second, external sources of lead exposure, especially the dietary intake of lead, was not considered in the current analysis. Food contamination in food source of dietary sodium and potassium may also provide possible explanations for the distinct relationships between bone lead and urinary lead in different Na/K ratio groups. However, there is no compelling evidence to support that dietary intake of lead contributes to elevated lead body burden (e.g., as shown in a recent study conducted in the National Health and Nutrition Examination Survey) [62]. The sources of external exposure to lead may also include air, contaminated drinking water, and older housing stocks with lead-based paint [63,64,65]. Our study was limited by the fact that lead from such external sources was not measured and we were unable to distinguish between lead from the skeleton and lead from external sources. Third, 24-h urine samples were collected only once in our study. The sodium and potassium intake based on this measure at a single time point would not capture the possible variation in micronutrient intake due to dietary changes over time, although rapid diet changes have not been observed in our previous studies in the NAS cohort [32,33]. Fourth, the timing of bone lead measurements was between 1991 and 2002. There has been a continuous decreasing trend in blood lead concentrations in the U.S. population over the last two decades [66]. However, it should be noted that unlike blood lead, with a half-life of approximately 30 days, lead in the bone has a half-life ranging from years to decades [16]. Updated evidence using the NAS data showed a slow decline of bone lead concentrations, indicating long-lasting lead exposure as mobilization from bone continues for decades after exogenous exposure declines [16]. Finally, participants in our study were predominantly white, middle-aged to older men, 95% of whom were of European descent, thus the generalizability of our findings could be limited. Future studies are needed to replicate our findings in women and other racial/ethnic groups.

## 5. Conclusions

Our findings suggest that a high intake of sodium relative to potassium may increase bone lead resorption among the middle-aged to older men. In this context, the release of bone lead into the circulation may be minimized by dietary recommendations that include both decreased sodium and increased potassium intakes. However, our findings need to be interpreted with a caution given the modest sample size. More epidemiologic studies utilizing longitudinal design with larger sample sizes as well as animal studies directly evaluating the role of sodium, potassium, and other dietary nutrient intake in bone lead resorption are needed to confirm our findings. If confirmed, such information will be crucial to help prevent lead toxicity, especially in populations where mobilization of lead from the bone is the dominant contributor to lead body burden. These findings also provide the impetus to further investigate the relationship between bone lead and health outcomes, with the addition of dietary sodium/potassium intake as a potential modifier in future studies.

## Figures and Tables

**Table 1:** Characteristics according to 24-h urinary sodium-to-potassium ratio in the Normative Aging Study (*n* = 318).

Characteristics	Total Population (*n* = 318)	Tertiles of 24-h Urinary Na/K Ratio			
		Low: <2.2 (*n* = 106)	Medium: 2.2–3.0 (*n* = 106)	High: >3.0 (*n* = 106)	
	Mean ± SD	Mean ± SD	Mean ± SD	Mean ± SD	*p*-value ^1^
Age, years	66.6 ± 7.0	66.9 ± 7.3	67.1 ± 6.7	65.8 ± 7.0	0.33
BMI, kg/m^2^	27.7 ± 3.9	26.6 ± 3.5	28.2 ± 4.2	28.2 ± 3.8	0.003
Urinary lead ^2^ (μg/day)	4.9 (1.8)	4.6 (1.8)	5.0 (2.0)	4.9 (1.7)	0.60
Patella lead ^2^ (μg/g)	30.6 (1.8)	27.8 (1.8)	33.9 (1.7)	30.4 (1.8)	0.04
Tibia lead ^2^ (μg/g)	19.8 (1.9)	18.6 (1.8)	21.7 (1.8)	19.4 (2.0)	0.18
Blood lead ^2^ (μg/dL)	5.5 (2.0)	5.0 (2.1)	5.7 (1.9)	6.0 (1.8)	0.10
Urinary sodium (mmol/day)	133 ± 61.2	99.5 ± 40.3	143 ± 56.6	158 ± 68.0	<0.0001
Urinary potassium (mmol/day)	53.0 ± 23.1	61.3 ± 24.5	55.7 ± 22.1	42.2 ± 17.9	<0.0001
Urinary Na/K ratio	2.7 ± 1.1	1.7 ± 0.4	2.6 ± 0.2	3.8 ± 0.8	<0.0001
Serum creatinine (mg/dL)	1.2 ± 0.2	1.2 ± 0.2	1.2 ± 0.1	1.2 ± 0.2	0.10
Urinary creatinine (mg/dL)	84.9 ± 44.2	76.8 ± 41.8	84.6 ± 42.4	93.3 ± 47.1	0.03
Creatinine clearance rate (mL/min)	71.1 ± 32.5	68.5 ± 33.9	70.1 ± 29.7	74.6 ± 33.8	0.37
Urinary N-telopeptide (nM BCE/mM creatinine)	54.9 ± 47.4	55.9 ± 38.8	56.9 ± 65.4	51.9 ± 30.8	0.73
Dietary calcium intake (mg/day)	826 ± 451	961 ± 482	770 ± 488	747 ± 342	0.0007
Dietary vitamin C intake (mg/day)	166 ± 101	195 ± 127	157 ± 95	144 ± 65	0.0007
Total energy intake (kCal/day)	2027 ± 896	2147 ± 840	1932 ± 1055	2003 ± 762	0.21
Smoking status ^3^					<0.0001
Never smoker	93 (29.2%)	32 (30.2%)	31 (29.3%)	30 (28.3%)	
Former smoker	194 (61.0%)	66 (62.3%)	63 (59.4%)	65 (61.3%)	
Current smoker	31 (9.8%)	8 (7.6%)	12 (11.3%)	11 (10.4%)	

^1^*p*-values from ANOVA for all continuous variables (urinary lead, patella lead, tibia lead, and blood lead were log-transformed). *p*-value from the Fisher-exact test for smoking status. ^2^ Geometric mean (geometric standard deviation) were calculated for urinary lead, patella lead, tibia lead, and blood lead concentrations. ^3^ N (%) was shown for smoking status.

## Supplementary Materials

The following are available online at https://www.mdpi.com/2072-6643/11/11/2750/s1, Figure S1: Percent change in 24-h urinary lead excretion (µg/24 h) per 2-fold increase in patella and tibia lead concentrations, stratified by tertiles of 24-h sodium-to-potassium (Na/K) ratio, after adjusting for blood lead concentrations, Figure S2: Percent change in 24-h urinary lead excretion (µg/24 h) per 2-fold increase in patella and tibia lead concentrations, stratified by tertiles of 24-h sodium-to-potassium (Na/K) ratio, after adjusting for interaction terms of NTx with patella and tibia lead, respectively.
