# Supplementary material for: Effect of Dietary Sodium and Potassium Intake on the Mobilization of Bone Lead among Middle-Aged and Older Men: The Veterans Affairs Normative Aging Study
Source: Nutrients. 2019 Nov 13;11(11):2750. doi: 10.3390/nu11112750 (PMC6893449; doi:10.3390/nu11112750)
Supplement: Supplementary file 1 [file nutrients-11-02750-s001.pdf]

Supplementary Materials:

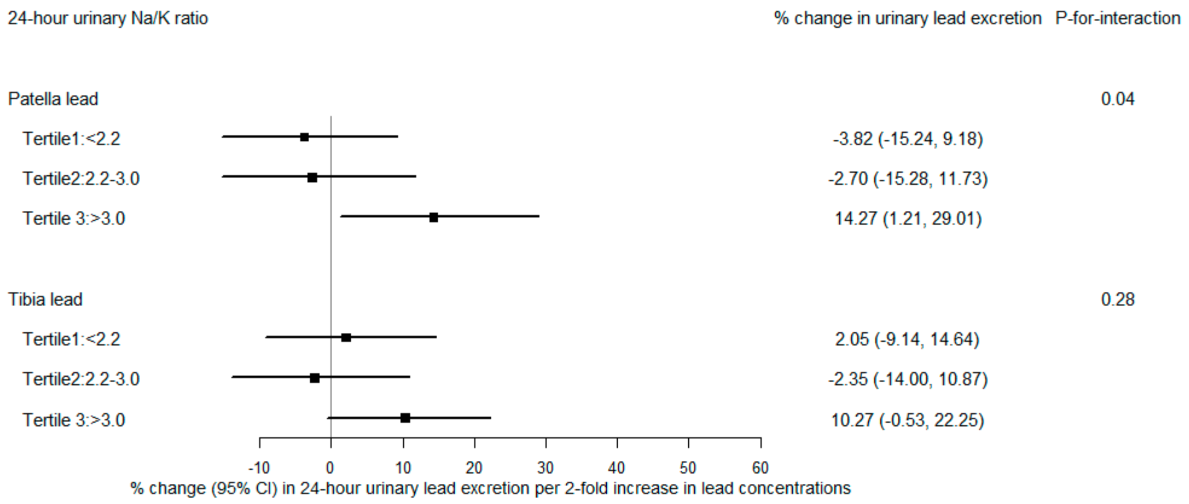

Supplemental Figure 1. Percent change in 24-hour urinary lead excretion (µg/24h) per 2-fold increase in patella and tibia lead concentrations, stratified by tertiles of 24-hour sodium-to-potassium (Na/K) ratio, after adjusting for blood lead concentrations. All models were adjusted for age, body mass index, smoking, vitamin C intake, calcium intake, total energy intake, and blood lead concentration.

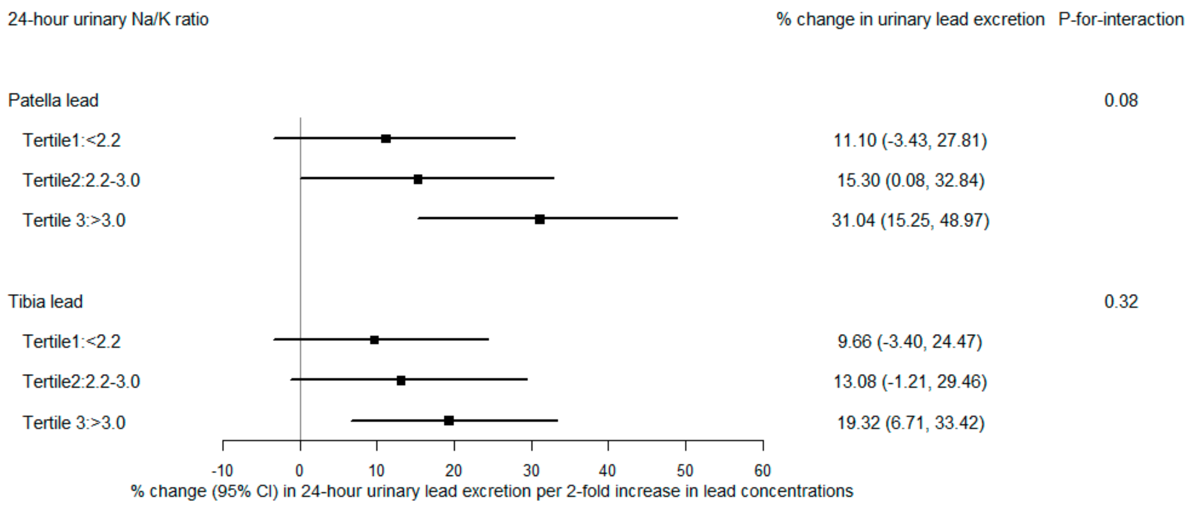

Supplemental Figure 2. Percent change in 24-hour urinary lead excretion (μg/24h) per 2-fold increase in patella and tibia lead concentrations, stratified by tertiles of 24-hour sodium-to-potassium (Na/K) ratio, after adjusting for interaction terms of NTx with patella and tibia lead, respectively. All models were adjusted for age, body mass index, smoking, vitamin C intake, calcium intake, and total energy intake.
